# Exploring the Differential Effects of Online Reviews on Film's Box-Office Success: Source Identity and Brand Equity From an Integrated Perspective

**DOI:** 10.3389/fpsyg.2020.00217

**Published:** 2020-02-21

**Authors:** Kai Zhao, Xiaocong Yang, Xiaobo Tao, Xiaoyu Xu, Jinkai Zhao

**Affiliations:** ^1^School of Economics and Finance, Xi'an Jiaotong University, Xi'an, China; ^2^School of Public Administration, Guangzhou University, Guangzhou, China; ^3^College of Economics and Management, North China University of Technology, Beijing, China; ^4^College of Economics and Management, Shandong University of Science and Technology, Qingdao, Shandong, China

**Keywords:** online reviews, source identity, brand equity, verified users, unverified users

## Abstract

This study investigates the differential effects of online reviews on actual sales in cases where information regarding source identity and brand equity is accessible. The data were collected from an influential online film review platform in China. Two distinctive features of this study are: (1) source identity is expressed as “verified user” or “unverified user” according to posters' ticket payment status and (2) the interactive effect between source identity and brand equity on box-office success is examined. Using econometric estimations, the results reveal the following: (1) the positive effect of verified users' online review valences on the number of tickets purchased for films decreases in association with high brand strength; (2) the variance of verified users' online reviews positively affects the number of tickets purchased for films with high brand strength, but such an effect is negative with low brand strength; (3) the variance of unverified users' online reviews positively influences the number of tickets purchased for films with low brand strength, but it negatively influences the number of tickets purchased for films with high brand strength. Thus, these findings suggest that it is better for business leaders to understand not only why producers of online reviews are satisfied or dissatisfied, but also how consumers interpret and interact with different types of online reviews and which are important. This requires a smart and flexible collaboration among different business units within film company.

## Introduction

On-going discussion regarding the role of online reviews has exerted profound influence on theoretical development in the fields of marketing, information systems, and psychology, marking the beginning of a widening stream of interdisciplinary work on the concept of online customer reviews (OCRs; King et al., 2014). However, the proliferation of fake reviews presents new complications regarding the role of OCRs within the decision-making process of consumers. Apparently, it is not easy to tell whether a review is true or fake. The rapid progress of AI technology has made the boundary between authentic and fake online reviews increasingly obscure. Even though considerable studies have designed or developed various automated methods to distinguish the authenticity of online reviews, regular detection of fake reviews can be very expensive for large service enterprises and even worse, consumers are still inevitably exposed to unreliable information (Chen et al., 2013).

For issues listed above, the present study puts forward a creative thinking of online environment management pattern. It is important to state at the outset that our analysis does not focus on examining the tracking of spammers' rating behavior (e.g., untruthful reviews, brand-only reviews, and non-reviews; Jindal and Liu, 2008). Rather, we view unreliable online reviews as heuristic cues and attempt to figure out how they affect consumers' product perceptions and purchase behaviors. Specifically, we seek appropriate answers to the following questions: (a) what are the effects of different online reviews on consumers' behaviors of buying movie tickets online, and (b) how can other important marketing communication factors moderate such effects.

For this study, the data of reviews were collected from a Chinese movie ticketing website Gewara.com, on which the online traffic monitoring information, including volumes, valences, and variances of movies for sale, as well as the information regarding movie features is released. In addition, to ensure the quality of the online information, only paying users can post online reviews or ratings of the movies they have paid for, labeled as “verified,” whereas other users' ratings and reviews are labeled as “unverified.” By combining the information with the matching data of box-office sales collected from cbooo.cn^1^, we modeled the box-office as a function of influential factors, including the total number of online reviews, overall valence and total rating variance. As the online communication environment is extremely complex, a novel measure is proposed to elucidate the differences between the effects of “real” user and “fake” online reviews on consumers' purchase decisions; overall valence and total rating variance are decomposed into verified user and unverified user subcategories, namely valence of verified users' reviews, valence of unverified users' reviews, variance of verified users' reviews, and variance of unverified users' reviews. Finally, concerning the underlying relationships between different marketing factors, we believe that brand equity and source identity change consumers' purchase behaviors through a variety of mechanisms. Therefore, the interaction terms of brand strength with verified users' or unverified users' online review valences and verified users' or unverified users' variances are included into the model.

This study has three major contributions to the related studies on psychology and marketing. First, fake online review is an emerging issue, but there are still unclear aspects in determining how it affects sales and in determining the interactive effects of various marketing factors, such as different source identities and brand strength on consumer's purchase decision. Herein, several complex situations involving both source identity and brand equity are simulated, which enriches the research on factor complementariness in marketing communication. Second, most previous studies have focused on the western film industry, but few studies have explored the determinants of purchase intentions and behaviors of Chinese consumers. Given the cultural and social differences, the findings based on western backgrounds maybe not generalizable in China. Therefore, this paper expects to bring more insights into consumers' behaviors. Third, this study attempts to establish an alleviating mechanism instead of a prevention mechanism, arguing that verified or unverified online reviews, in association with the presence of brand equity, may have significantly different effects on consumers' purchase behaviors. This argument provides a new way to study the control of unreliable information, which is conducive to understanding and solving such a problem.

The rest of this paper is organized as follows. The conceptual frameworks and hypotheses developed are illustrated in section Conceptual Frameworks and Hypotheses Development. In section Econometric Model and Variable Settings, the econometric model is presented. The empirical results and theoretical and managerial implications are discussed in sections Results and Discussion and Conclusions, respectively.

## Conceptual Frameworks and Hypotheses Development

### Psychological Choices Model and Attribution Theory

Cognitive psychologists believe that subjective interpretation matters as dealing with online information has a nature of uncertainty (Gollwitzer and Kinney, 1989). When consumers read online product reviews posted by strangers, they must draw conclusions about the authenticity and integrity of these reviews. However, as the Hansen Model of Psychological Choice (Hansen, 1976) suggests, the interpretation of online information is a complex perceptual process which involves many environmental, contextual and cognitive factors. Therefore, the effectiveness of an online review appears to be determined by both consumers and the product itself. Movies are mass-consumption, and very popular experiential products, whose relevant attributes cannot be known until the trial or use of the product or service (Nelson, 1974), and moviegoers may have an expectation regarding film's quality but this expectation can be either fulfilled or not (Boor, 1992; Tsao, 2014). When deciding whether to go to a cinema to watch a film, evaluating cognitive cues online has always been a convenient way for consumers to form perceptions about this product. This mental process can be based on a direct self-evaluation toward movie product attributes, such as production costs, screen format, genre, budget size, and director or leading actors' market reputation; or consumers are more likely to collect indirect information from others (Larson and Denton, 2014). According to the attribution theory (Kelley, 1967), an online review can imply the effect of distinctiveness if it does not often appear (e.g., a picky reviewer posted a very positive review), the effect of consistency if such an attitude toward a movie does not change over time, and the effect of consensus if most critics consider that movie is good or bad. These effects can lead to consumers' attributional behaviors, i.e., consumer's own analysis regarding the reasons why critics made the judgments toward a movie. In other words, consumers' evaluations of a movie are more likely to follow the comments of film critics if the comments are distinctive, consistent and in line with the opinions of other reviewers (D'Astous and Touil, 1999).

Numerous studies have empirically measured critics' judgments in terms of the volume of reviews (Chevailier and Mayzlin, 2006), the valence of reviews (Liu, 2006), and the variance of reviews (Moe and Trusov, 2011). Even though these studies mainly focused on the determinants that affect the economic performance, namely, box-office sales of a given movie at the collective level, they still essentially explained how consumers' psychological choice when picking a certain film is influenced by various factors from the perspectives mentioned above. Therefore, the present study aims to reconcile some inconsistences of previous studies in the application of economic and psychological approaches. Specifically, we extend the existing research scope by linking consumers' behaviors with multi-dimensional online information with differential market outcomes.

### Brand Equity

Brand equity can be defined as “the differential effect that brand knowledge has on consumer's response to marketing activity” (Hoeffler and Keller, 2003, p. 421). The studies on branding have shown that products with strong brand equity have greater advertising power with which to increase consumers' attention (Feng et al., 2019), are more resistant to negative information (Dawar and Pillutla, 2000), and can be used as signals of product creditability (Erdem et al., 1999). These findings suggest that the occurrence of brand effect is closely related to uncertain circumstances when consumers lack sufficient information to identify favorable products.

OCRs (online customer reviews) can deliver signals to consumers coping with uncertainty (Lau et al., 2011; Wei et al., 2018). However, the OCR-provided signals derive from consumers' perceptions toward credibility and are independent of marketers' interests; thus, it is key to compare these with the effect of brand equity. Early studies attempted to understand how firms could signal when product quality was not easily observed. Cobranding is believed to be an effective strategy for reducing consumers' perceived risk with new products (Montgomery and Wernerfelt, 1992; Chiambaretto and Gurău, 2017). Follow-up studies have extended these discussions to the context of OCRs. With data from the video game industry (GameSpot.com), Zhu and Zhang (2010) investigated how the popularity of games moderates the influence of online reviews on sales. They stated that less popular games were more likely to be affected by OCRs. Ho-Dac et al. (2013) provided evidence for the effect of brand equity in the Blu-ray and mature DVD player industries, demonstrating that the sales of weak brands were positively related to the valence of OCRs, whereas OCRs had no noteworthy effect on sales of strong brands.

Despite the importance of understanding the influence of OCRs on product sales through accessing branding signal, it remains unclear how brand equity moderates the effect of OCRs in the film industry. Studies have employed data on various brand-related factors, such as director, cast, award nominations, movie popularity, and box-office history as proxies of “star power” (Elliott and Simmons, 2008; Fetscherin, 2010; Sun et al., 2014), but findings concerning the effect of star power on box-office sales are mixed. The specific effect of brand equity has also not been clearly separated from the effect of OCRs. Therefore, a discussion of the moderating effects of brand equity on the overall effectiveness of online reviews will follow.

### Social Norms and Social Identity

Apart from the aforementioned factors, anonymity has become a common problem with the rapid development of the Internet. In most cases, only a user's forum name, avatar, and user service grade are accessible on a review platform, meaning that their real name, biography, photo, or other information that could reveal true identity are not available. This phenomenon has led to concerns about source identity. A prospective customer could ask for advices from friends or family in order to reduce uncertainty, as the influence exerted by social norms that others approve or disapprove a particular behavior is important in determining consumers' actual behaviors (Ajzen, 1991). In an online environment, such a perception that a behavior is socially approved or disapproved can be also formed by consumers' online peers. In this case, consumers tend to approach social norms through certain behaviors (e.g., following other online posters' approved or disapproved actions) to gain a sense of group belonging so as to construct social identity (Ashforth and Mael, 1989).

Therefore, source identity seems to be particularly important for consumers' purchase intentions and behaviors in online communications from the perspective of social psychology. Studies have shown that identity exposure can lead to changes of consumer behaviors which affect online sales growth. For instance, Forman et al. (2008) advocated the importance of reviewer identity disclosure. Based on the data from Amazon, their findings support the idea that disclosure of reviewers' demographic information is associated with growth in subsequent online product sales. In an examination of the interactive effects of review valance and source identity in the tourism industry, Kusumasondjaja et al. (2012) found that OCRs become more credible when the reviewer's identity is disclosed. However, the role of information identity in the formation of consumer perception toward movie products is still less explored. Therefore, this study uses verified and unverified online reviews as two key variables to investigate consumers' reactions to comments of different qualities, which will shed some new lights on how the reviewer's identity information may affect consumers' perceptions of online reviews credibility and associated market outcomes.

### Hypotheses

**Figure 1:**
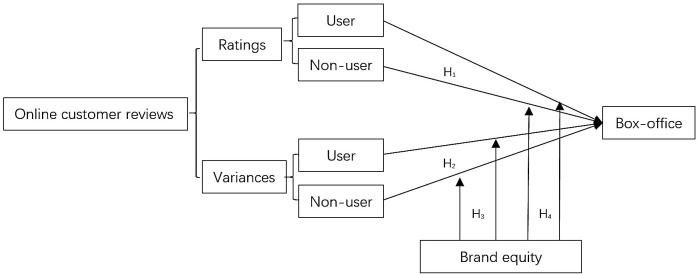
Analytical model of the effects of verified users' and unverified users' online reviews on box-office performance.

Although previous researches on online reviews have investigated the role of source identity and brand equity, few studies have considered their interactional effects. Therefore, the present study suggests that source identity and brand equity play crucial roles in forming consumers' perceptions of online review credibility and subsequent purchase behaviors and simulates this practical marketing environment. To this end, this study employs the psychological choice model (Hansen, 1976), in which the effectiveness of online reviews on consumers' purchase decisions is moderated by product characteristics (e.g., popularity or brand strength), consumer characteristics (e.g., verified/unverified identity), and external factors (e.g., competition and advertisement; see Figure 1). The research hypotheses we seek to address are as follows:

Hypothesis 1: the present study investigates the valence of online reviews that may lead to consumers' purchase intentions and behaviors. The theory behind measuring valence or consumer attitude is that positive comments will encourage other consumers to buy a product whereas negative comments will discourage them (Dellarocas et al., 2007). We collected detailed review information including the number of online reviews (i.e., volume) and the average ratings (i.e., valence; a higher scorer indicates a more positive attitude and vice versa) for each film. On this basis, the question of interest is whether the valence (i.e., an averaged rating) of verified online reviews has a larger effect on the box-office sales of a film than that of unverified users. According to the theory of IAM (information adoption model), source credibility has a certain effect on forming consumer's perception regarding information usefulness and whether the information can be adopted (Erkan and Evans, 2016). Therefore, an arithmetic mean of ratings posted, labeled as “verified,” is likely to provide visual linguistic cues (either positive or negative) for consumers to build trust and thus influence their purchase intentions and behaviors. In comparison, even though consumers are less accurate at identifying unreliable or fake reviews (Jindal and Liu, 2008), many consumers still tend to be skeptical of online reviews from unverified users because they think the motives behind those reviews are questionable. Thus, the following hypothesis (H1) is proposed:

*H1: Verified online reviews (valence) have a stronger positive effect on the box-office sales of a film than those of unverified users (valence)*.

It is worth mentioning that many people may also post-anonymous reviews because they want to criticize a movie without running the risk of backlash (Dellarocas and Wood, 2008). If that is true, some unverified reviews can be also deemed to be truthful thus the validity of this taxonomy (i.e., verified vs. unverified reviews) may be questionable from the perspective of “information authenticity.” However, the present study aims to measure how consumers' psychological choices shift toward “heuristic” cues. According to the heuristic–systematic model (HSM; Chen and Chaiken, 1999), consumers process information through two distinct approaches: systematic information processing—in which recipients' attitudes toward information are influenced by the content of the information—and heuristic information processing—in which recipients focus on the situational heuristic cues of the information (including its source, format, length, etc.). Compared with heuristic information processing, systematic information processing requires more cognitive resources from consumers, such as time, perceived risks and skill level (Chaiken, 1980). The HSM recognizes that not all decisions are worthy of particular efforts to generate high accuracy, as consumers adjust their decision-making process based on their perceptions of importance, risks, time, and other cognitive factors (Zhang et al., 2012). In this case, when consumers encounter a high number of online reviews, their cognitive capacity levels are overwhelmed by systematic processing, and consumers tend to further capitalize on their assessment of information providers as an easy and convenient cognitive shortcut (i.e., heuristic cues) to help them reach evaluative conclusions (i.e., the sufficiency threshold) and take appropriate actions (Forman et al., 2008).

Therefore, a measure to elucidate the differences between the effects of verified and unverified online reviews on consumers' purchase decisions is more likely to reflect the true psychological state of consumers toward different information sources. In comparison, it is a subjective and challenging task for consumers to identify the authenticity of reviews by examining related content due to information overload or because other product information is not accessible. Moreover, technically on Gewara, a reviewer will be permanently marked as “verified,” as long as he/she purchased a ticket of a given movie and that ticket can be only used in Gewara-franchised cinemas. This is a straightforward “purchase-watch-review” process, similarly to how the online review system operates on ebay. Thus, even reviewers wish to anonymously publish their reviews to avoid backlash, their comments will be always marked as “verified.” The rationality of categorizing online reviews as “verified” and “unverified” by Gewara is: (1) most of verified reviews are tickets purchasers who watched a given movie in one of Gewara-franchised cinemas; (2) there is no chance for a reviewer who watched a movie and posted a comment without exposing identity. It has to be admitted that in some extreme cases, a consumer may watched a movie somewhere else, and came to Gewara to post reviews anonymously, but to our best knowledge, there is no research that has attempted to analyse such a very proactive behavior based on the existing data generation method, and this is also very less likely to occur among Chinese consumers, as they do not behave so proactively to express ideas under a collectivistic culture (e.g., Fang et al., 2013). Therefore, even such a behavior does exist, but its impact on consumer's cognitive process toward different online information is very limited, and ratings from verified vs. unverified reviews would not have their origins in being qualitatively different.

Hypothesis 2: The variance of online reviews (i.e., the range of opinion differences) reflects the heterogeneity of consumer evaluations. A significant body of research has shown that the effect of a variance of online reviews on consumers' purchase behaviors is more likely to be context-specific compared to that of valence. For instance, a higher variance maybe associated with lower product sales if consumers exhibit risk aversion behaviors (Zhang and Dellarocas, 2006). However, it can be also associated with higher product sales, as a high variance in online product reviews may arouse consumers' curiosity, consequently stimulating consumers' intentions to buy the product (Martin et al., 2007), or signify highly extensive product information (Godes and Mayzlin, 2004; Clemons et al., 2006). These findings present solid evidence that the effect of a variance can be moderated by factors, such as consumer traits or product features.

This study further reconceptualizes variance as a “differential” rather than a “context-specific” eWOM, to broaden the scope of its construct and to study its influence on consumer behaviors. Specifically, we clarify that variance has a “unverified” feature for consumers to form perceptions toward online information which in turn acts on purchase behaviors based on two scenarios. In the first scenario, a group of paid anonymous posters deliberately raise the rating of a film. Certain consumers may become aware of this strategy and respond by intentionally leaving negative spam reviews, thereby widening the variance of the reviews. This interaction unconsciously enhances public awareness of the film and thus increase the number of ticket purchased. This situation can be deemed realistic for two reasons: first, the major issue for eWOMs comes from the challenges of dealing with source identities. As product information created by consumers is now believed to have a greater influence on consumer decisions than that traditionally created by sellers, firms look for fair and creditable recommendations of their products from online users who post and share their product experiences (King et al., 2014). However, the openness of eWOMs means that posting fraudulent reviews online only comes with low social and economic costs. The real “pusher” is difficult to be traced even a fake review has been identified, thus many firms have strategically manipulated online reviews in order to influence consumers' purchase decisions (Dellarocas, 2006). So, it is no surprise that posting online consumer reviews in exchange for payment has become increasingly common and organized recently (Streitfeld, 2012). Second, consumers do sometimes attempt to protect online review forums from abuse by posting reviews they believe is falsified (Larson and Denton, 2014). Chaudhuri (2000) states that a “psychological risk” of consumers refers to the emotional inconsistency between the products they purchased and self-images. In this case, reading through unusual positive or negative comments may elicit a threat to the ego of consumers, which further increases the negative self-evaluation and the possibility of spreading these feelings to others.

In the second scenario, a “positive/negative opinion spam,” as mentioned in the first situation, may signify an incentive to a rival film promotion company to choose a distinctive strategy. This behavior should produce a “separating equilibrium” (Boulding and Kirmani, 1993): one promotion company hires anonymous posters to raise the rating so as to receive higher market compensation; and instead of adopting the same strategy, namely, increasing the rating of its own film in a same release schedule, the other promotion company would, on the contrary, hire paid anonymous posters to lower the film rating of its rival, further allowing consumers to distinguish films based on a “credible signal” (Spence, 1977). That's because a decrease of a competitor's film rating is likely to highlight the rating of its own film. This phenomenon is quite common in the Chinese film market. For instance, the director of “King's Banquet” admitted that anonymous posters were purposely hired to increase the overall rating of the film, as he believed that the initial appearance of a large number of negative reviews in a very short time is a manipulation by rivals. This may widen the variance of the reviews for a given movie and also cause consumers to take actions, such as posting “watchdog comments” to warn other consumers of this strategy (Trope and Liberman, 2010), thus altering the public interpretation of the original perception toward the film and significantly influencing the overall ticket sales in a long run.

Based on the above demonstration, we hypothesize and explore the differences in the influence of the verified vs. unverified dimensions of variance. We assume that a unverified variance, beyond the context-specific scope, is likely to be more mass-public oriented, and the attached cognitive cues may cause consumers to become more prosecutorial and impulsive in aggressing against perceived wrongdoers (Larson and Denton, 2014), thus, moderating the relative magnitudes of consumers' risk aversion and curiosity more dramatically (Cheung and Thadani, 2012). Therefore, it is predicted that the presence of unverified variance of ratings is more likely to increase the popularity of a film, and the following hypothesis (H2) is proposed:

*H2: The effect of verified users' online review variance on increasing the box-office sales of a film is lower than that of unverified users*.

Hypotheses 3(a) and 3(b): Brand strength refers to the extent of association between brand awareness and positive brand image (Keller, 1993). Products with high visibility and prestige exhibit considerable brand strength (Levin et al., 1997). Therefore, star power can be used as a proxy variable for product brand strength. When a film's actors or director have won awards for best actor or director at worldwide film events, such as the Golden Rooster Awards, Hong Kong Film Awards, Golden Horse Film Festival and Awards, and Academy Awards, the star power of the film increases, and its corresponding brand strength is promoted (Elberse, 2007).

As heuristic cues, brand strength and star power alleviate the effect of verified users' online reviews on product sales. People tend to believe that the quality of a film is guaranteed if its actors or director have won film awards; in that case, potential consumers are less reliant on the cues from verified users' online reviews and film quality ratings. Accordingly, when the brand strength or star power of a product is substantial, the effect of verified users' online reviews on its sales is reduced. However, when no heuristic cue is available for reference, verified users' online ratings become a crucial cue for film quality; a high overall buyer review rating will encourage potential consumers to purchase tickets (Nelson and Glotfelty, 2012).

Consumers have limited trust toward unverified review ratings; however, consumers' positive impressions and appraisal toward a film are increased when the film exhibits considerable brand strength or star power, enhancing consumers' purchase behaviors (Rosen, 1981). When a film exhibits low brand strength or star power, unrealistically positive ratings from unverified users may prompt consumers to react negatively to the film and avoid watching it (Hamlen, 1994). Hence, the following hypotheses (H3) are proposed:

*H3: The brand strength of a product moderates the effects of verified users' and unverified users' online reviews (valence) on its sales*.

*H3a: Verified users' online reviews (valence) have a higher effect on the sales of a product with low brand strength (e.g., a film with weak star power) than on the sales of a product with high brand strength (e.g., a film with strong star power)*.

*H3b: Unverified users' online reviews (valence) have a lower effect on the sales of a product with low brand strength (e.g., a film with weak star power) but not that of a product with high brand strength (e.g., a film with strong star power)*.

Hypotheses 4(a) and 4(b): When a film exhibits strong star power, potential consumers' risk perceptions—which are induced by the high variance of verified users' reviews—are reduced, and consumer curiosity is increased. Therefore, a high review variance is likely to increase the box-office sales of a film. Conversely, because a film with weak star power provides no reliable cues on its quality, a high variance in verified user reviews substantially increases consumers' risk perceptions and influences their decisions, thus negatively affecting the film's sales.

When the variance in unverified users' reviews toward a film with strong star power is high, potential customers may consider positive reviews as sensationalism by filmmaking or promotion companies and negative reviews as trolling by other consumers, thereby increasing consumers' risk perceptions and lowering box-office sales. For a film with weak star power, potential consumers may regard the film as lacking the economic strength to hire paid anonymous posters, so they may assume that the high variance in unverified reviews is resulted from the reactions of consumers with varying preferences; thus, some consumers may become curious and purchase tickets for the film (West and Broniarczyk, 1998; Sun, 2012). Accordingly, the following hypotheses (H4) are proposed:

*H4: A product's brand strength moderates the effect of variance in verified users' and unverified users' online reviews on the product's sales*.

*H4a: The variance in verified users' online reviews has a lower effect on the sales of a product with low brand strength (e.g., a film with weak star power) than on the sales of a product with high brand strength (e.g., a film with strong star power)*.

*H4b: The variance in unverified users' online reviews has a lower effect on the sales of a product with high brand strength (e.g., a film with strong star power) than on the sales of a product with low brand strength (e.g., a film with weak star power)*.

## Econometric Model and Variable Settings

### Econometric Model

The following econometric Equations (1–3) are established to express the differences between the effects of verified users' and unverified users' online review ratings and variances on film sales.

(1) $$\begin{array}{l} lnbosales=\alpha_{0}+\alpha_{1}lnvol+\alpha_{2}Trate+\alpha_{3}Tvar+\alpha_{4}star\\ \;+\gamma Z+\varepsilon \end{array}$$

(2) $$\begin{array}{l} lnbosales=\alpha_{0}+\alpha_{1}lnvol+\alpha_{2}RURs+\alpha_{3}RNURs+\alpha_{4}Tvar\\ \;+\alpha_{5}star+\gamma Z+\varepsilon \end{array}$$

(3) $$\begin{array}{l} lnbosales=\alpha_{0}+\alpha_{1}lnvol+\alpha_{2}Trate+\alpha_{3}VURs+\alpha_{4}VNURs\\ \;+\alpha_{5}star+\gamma Z+\varepsilon \end{array}$$

where *lnbosales* is the logarithm of online sales on Gewara, *lnvol* is the logarithm of the total number of online reviews, *Trate* is the total valence, *RURs* is the valence of verified users' reviews, *RNURs* is the valence of unverified users' reviews, *Tvar*, *VURs*, and *VNURs* are the total variance of ratings, variance of verified users' reviews, and variance of unverified users' reviews, respectively; *star* is a dummy variable for product brand strength (0 = weak; 1 = strong)^2^, γ*Z* is the observable vector of the control variables, excluding the number of reviews, and ε is an error term.

To examine the moderating effect of brand strength (star) on the relationship between online reviews and sales, the interaction terms of brand strength and online review ratings and variances are included into Equations (4, 5), as shown below:

(4) $$\begin{array}{l} lnbosales=\alpha_{0}+\alpha_{1}lnvol+\alpha_{2}Tvar+\alpha_{3}RURs+\alpha_{4}RNURs\\ \;+\alpha_{5}RURs^{*}star+\alpha_{6}RNURs^{*}star+\alpha_{7}star\\ \;+\gamma Z+\varepsilon \end{array}$$

(5) $$\begin{array}{l} lnbosales=\alpha_{0}+\alpha_{1}lnvol+\alpha_{2}Trate+\alpha_{3}VURs+\alpha_{4}VNURs\\ \;+\alpha_{5}VURs^{*}star+\;\alpha_{6}VNURs^{*}star+\alpha_{7}star\\ \;+\gamma Z+\varepsilon \end{array}$$

For endogeneity concerning Internet media reviews variables in the decision-making process, a simultaneous equation model is further introduced. Compared to Holbrook and Addis (2008) and Peng et al. (2019), who defined the possible reverse causality between box-office revenues and online reviews, our model setup is more complicated as it involves box-office revenues, variance, valence and their secondary variables (e.g., verified users' variance and unverified users' variance). In order to test the sensitivity of our results while maintaining the basic requirements of simultaneous equation modeling, such as identification conditions, we use different variable combinations to construct the simultaneous equation sets for Equations (1–5) with respect to the potential endogeneity issue.

(6) $$\begin{array}{l} lnbosales=\alpha_{0}+\alpha_{1}lnvol+\alpha_{2}Tvar+\alpha_{3}RURs\\ \;+\alpha_{4}RNURs+\alpha_{5}RURs^{*}star+\alpha_{6}RNURs^{*}star\\ \;+\alpha_{7}star+\alpha_{8}Sequel+\alpha_{8}Movie\;type\\ \;+\alpha_{9}Length+\varepsilon \end{array}$$

(7) $$\begin{array}{l} lnvol=f_{0}+f_{1}lnbosales+f_{2}Trate+f_{3}Newweek\\ \;+f_{4}Holiday+f_{5}format\;type+f_{6}Foreign\;film+\psi \end{array}$$

(8) $$\begin{array}{l} Tvar=e_{0}+e_{1}Trate+e_{2}Foreign\;film\\ \;+e_{3}Ownership+e_{4}Actress{+e}_{5}lnbosales+\zeta \end{array}$$

(9) $$\begin{array}{l} RURs=g_{0}+g_{1}lnbosales+g_{2}VURs+\upsilon \end{array}$$

(10) $$\begin{array}{l} RNURs=s_{0}+s_{1}lnbosales+s_{2}VNURs+\delta \end{array}$$

(11) $$\begin{array}{l} RURs^{*}Starpower=p_{0}+p_{1}lnbosales+p_{2}star+p_{3}RURs+\omega \end{array}$$

(12) $$\begin{array}{l} RNURs^{*}Starpower=\kappa_{0}+\kappa_{1}lnbosales+\kappa_{1}star+\kappa_{1}RNURs+\nu \end{array}$$

Taking Equation (4) as an example, its simultaneous equation set (Equations 6–12) concerns the possibility of reverse causality between online sales and (1) the number of online reviews, (2) total variance, (3) valence of verified users' reviews, and (4) valence of unverified users' reviews. The interactive terms (5) valence of verified users' reviews × brand strength and (6) valence of unverified users' reviews × brand strength are also considered as endogenous as valence of verified users' reviews and valence of unverified users' reviews are pre-determined endogenous. For Equation (5), the reverse causality further relates to (7) total valence (8) variance of verified users' reviews, (9) variance of unverified users' reviews and the interactive terms, (10) variance of verified users' reviews × brand strength and (11) variance of unverified users' reviews × brand strength. More details are discussed in the section of robustness test.

### Data and Variable Settings

#### Sample Collection and Data Reprocess

The data used in this paper are mainly from Gewara^3^ and the China Box-Office^4^, which collected and adapted information of 308 movies released from January 2013 to October 2014 in China^5^. Gewara enables potential consumers to access the main information of a movie, such as cast, director, language, and release date, which helps consumers to develop their perceptions of product features and brand strength. The number of online reviews, namely, volume, is also observable and associated with the overall rating of a movie. The Gewara system has defined verified and unverified users and their corresponding activities in a clear way: if a user did not purchase a ticket on Gewara, all his/her activities on Gewara will be marked as “unverified”; otherwise are “verified.” The system also accounts for the statistics of verified and unverified users separately, which is convenient for researchers to collect. Both verified and unverified users can use a scale ranging from 1 to 10 to express their attitudes, 10 being the best and 1 being the worst. A window of summary statistics displays and compares the proportions of verified/unverified users who rated 9–10 points, 8–9 points, 6–8 points, 4–6 points, and 1–4 points, respectively, for a movie in the total number of users through a bar chart. As scores of verified users and unverified users are displayed separately in orange and gray, it is easy for consumers to form a straightforward impression of the score distribution (i.e., variance) and the weighted average score (i.e., valence) of verified and unverified users. Moreover, consumers can also develop their perception of information creditability through accessing individual verified and unverified users' ratings toward a particular movie in the comments section. For example, readers can classify the ratings posted by verified users and unverified user, and red marks indicate verified users. We believe that the review moderation system adopted by Gewara is effective in providing an appropriate online environment for consumers to interpret OCRs together with brand equity and source identity.

Descriptive statistics for all variables and constructs are presented in Table 1^6^. Particularly, it is worth mentioning that the number of unverified reviews for each point of rating scale of valence is large. This implies that the effect of unverified reviews is indeed substantial. We begin with a variable measurement and then the discussion of the empirical model. As detailed in the following sections, our main measures of the constructs are derived from the aforementioned online data, in addition to other industry sources available on the Internet. We define each measure and provide a brief rationale where appropriate:

**Table 1:** Variable statistics description.

**Dependent variable (and proxies)**	
Online sales	
Mean (SD)	117379.48 (215400.51)
Min, Max	149.0, 1994703.0
Total box-office	
Mean (SD)	13404.78 (22464.18)
Min, Max	9.0, 197893.0
Number of online like	
Mean (SD)	33636.48 (60341.97)
Min, Max	123.0, 554284.0
Number of online follows	
Mean (SD)	281345.62 (426958.55)
Min, Max	8166.0, 4187466.0
Ticket conversion rate	
Mean (SD)	0.26 (0.16)
Min, Max	0.0, 0.6
**Key independent variables**	
Total number of online customer reviews	
Mean (SD)	2279.98 (3493.87)
Min, Max	18.0, 23392.0
Total valence	
Mean (SD)	7.05 (1.25)
Min, Max	2.1, 9.3
Total variance of valences	
Mean (SD)	1.93 (0.46)
Min, Max	0.9, 3.0
Valence of verified users' reviews (RURs)	
Mean (SD)	7.28 (1.29)
Min, Max	3.1, 9.2
Total num. of reviews in rating 9–10^a^	139, 411
Total num. of reviews in rating 8–9	85, 090
Total num. of reviews in rating 6–8	83, 407
Total num. of reviews in rating 4–6	22, 400
Total num. of reviews in rating 1-4	20, 811
Valence of unverified users' reviews (RNURs)	
Mean (SD)	7.37 (1.17)
Min, Max	3.1, 9.3
Total num. of reviews in rating 9–10	153, 266
Total num. of reviews in rating 8–9	68, 090
Total num. of reviews in rating 6–8	87, 596
Total num. of reviews in rating 4–6	17, 269
Total num. of reviews in rating 1–4	24, 714
Variance of verified users' reviews (VURs)	
Mean (SD)	1.81 (0.48)
Min, Max	0.5, 3.2
Variance of unverified users' reviews (VNURs)	
Mean (SD)	1.98 (0.49)
Min, Max	0.8, 3.0
**Control variables**	
Length of the film	
Mean (SD)	103.34 (16.59)
Min, Max	75.0, 178.0
Number of films in the same week	
Mean (SD)	4.56 (1.78)
Min, Max	1.0, 9.0
Star power	101 (32.8%)
Follow-up Sequel	43 (14.0%)
Released in holiday	66 (21.4%)
Country of origin	
Domestic film	160 (51.9%)
Foreign film	93 (30.2%)
Co-production film	55 (17.9%)
Publisher	
State-owned sector	159 (51.6%)
Large private-sector	54 (17.5%)
Small private-sector	95 (30.8%)
Cinematic genre	
Story	96 (31.2%)
Comedy	61 (19.8%)
Actioner	54 (17.5%)
Romance	25 (8.1%)
Cartoon	31 (10.1%)
Horror	18 (5.8%)
Others	23 (7.5%)
Screening format	
2D	243 (78.9%)
3D	91 (29.5%)
IMAX	47 (15.3%)
Total (*N* = 308)	

*Source: Gewara and China Box-Office*.

a *The business perspective of setting up a cut-off criterion is quite different from researchers'. Ideally, researchers would like to see very detailed categories (i.e., 1; 2; 3; 4 … 10), however, online sellers intend to downplay the negative effect of low ratings, thus categorizing low ratings roughly (i.e., 1–4) while elaborately exhibiting high ratings (i.e., 8–9; 9–10) would be an effective strategy. We can see this is a primary strategy implemented by online sellers for managing online information, and this cut-off criterion does not produce biased results from calculating verified/unverified users' valences and variances, as this is the raw online information consumers can view and all their subsequent interpretations, reactions, and behaviors are based on this*.

(i) the proportions of verified users who rated 9–10 points, 8–9 points, 6–8 points, 4–6 points, and 1–4 points toward a given movie over the total number of users (i.e., verified and unverified users) in each grade of ratings can be expressed as gg5 = g5/(g5 + b5), gg4 = g4/(g4 + b4), gg3 = g3/(g3 + b3), gg2 = g2/(g2 + b2), and gg1 = g1/(g1 + b1), respectively, where g5, g4, g3, g2, and g1 are the number of verified users in each grade of ratings and b5, b4, b3, b2, and b1 are the number of unverified users in each grade of ratings, respectively. Similarly, the proportions of unverified users who rated a movie in each grade can be expressed, such as bg5 = b5/(g5 + b5).

(ii) the proportion of reviews posted by verified users in the total number of reviews can be expressed as ggbl = p5^*^gg5 + p4^*^gg4 + p3^*^gg3 + p2^*^gg2 + p1^*^gg1, where p5, p4, p3, p2, and p1 are the proportions of the number of reviews in each grade of ratings, respectively; and the proportions of the number of reviews posted by unverified users in the total number of reviews can be expressed as bgbl = p5^*^bg5 + p4^*^bg4 + p3^*^bg3 + p2^*^bg2 + p1^*^bg1.

(iii) the proportion of the number of verified users who rated 9–10 points in the total number of verified reviews can be expressed as pg5 = p5^*^gg5/ggbl and in other grades of ratings the formulas are pg4 = p4^*^gg4/ggbl, pg3 = p3^*^gg3/ggbl, pg2 = p2^*^gg2/ggbl and pg1 = p1^*^gg1/ggbl. Similarly, the proportion of the number of unverified users who rates 9–10 points in the total number of unverified reviews can be calculated as pb5 = p5^*^gb5/bgbl, and pb4, pb3, pb2, and pb1 are calculated in the same way.

(iv) the volume of online reviews posted by verified users can be expressed as sgg = gwl^*^ggbl, where gwl is the total number of online reviews for a given movie. Similarly, the volume of online reviews posted by unverified users is sbg = gwl^*^bgbl.

(v) the valence of online reviews posted by verified users can be expressed as rmean_gg = 9.5^*^pg5 + 8.5^*^pg4 + 7^*^pg3 + 5^*^pg2 + 2.5^*^pg1; the valence of online reviews posted by unverified users can be expressed as rmean_bg = 9.5^*^pb5 + 8.5^*^pb4 + 7^*^pb3 + 5^*^pb2 + 2.5^*^pb1. Here an average score is calculated for each grade of ratings, for instance, 9.5 = (9+10)/2.

(vi) the variance of online reviews posted by verified users can be expressed as [pg5^*^(rmean_gg −9.5)^2^ + pg4^*^(rmean_gg −8.5)^2^ + pg3^*^(rmean_gg −7)^2^ + pg2^*^(rmean_gg −5)^2^ + pg1^*^(rmean_gg −2.5)^2^]/(pg5 + pg4 + pg3 + pg2 + pg1); while the formula for unverified users is [pb5^*^(rmean_bg −9.5)^2^ + pb4^*^(rmean_bg −8.5)^2^ + pb3^*^(rmean_bg −7)^2^ + pb2^*^(rmean_bg −5)^2^ + pb1^*^(rmean_bg −2.5)^2^]/(pb5 + pb4 + pb3 + pb2 + pb1).

#### Dependent Variable

The dependent variable used in this study is the total number of online ticket sales from Gewara. In addition, for robustness tests, we also employed the box-office sales of the film (lnbox) from China Box-Office, the number of fans of the film (lnlike) from Gewara, the number of people following the film (lnatten) from Gewara, and the ticket conversion rate (trans = total number of ticket purchases ÷ total number of people paying attention). All dependent variables used in regression analysis except the ticket conversion rate are transformed using the logarithm function.

#### Independent Variables

Based on the above data generation process, the key independent variables in this study include total valences and total variances, online reviews form verified and unverified users (namely verified users' and unverified users' review valences and variances), and product brand strength, which involves the film's star power as the dummy variable (1 means that director or actors have won awards for best director or actor at representative film festivals, resulting in strong brand strength; and 0 means that director or actors have not won any award at film festivals, resulting in weak brand strength).

#### Control Variables

Regarding the control variables, the number of OCRs (i.e., volume), represents the total online reviews for each film on an online platform and is associated with the popularity effect of a film. The theory behind measuring volume is that the more popular a movie is, the more visitors and reviews it attracts. The other control variables on the characteristics of a film are its genre, its country of origin, whether it is a sequel (McKenzie, 2013), its screening format (Kim et al., 2017), the type of promotion (Ma et al., 2016), distribution company (McKenzie and Walls, 2013), and running time (in minutes) (Adams et al., 2000). Film genres is a control variable prevalently applied in the study of box-office sales (Liu, 2006), including drama, comedy, action, animation, romance, and thriller. As for a film's country of origin, it can be a domestic film, a foreign film, or a sino-foreign co-production. Sequel indicates whether the film is a sequel to a previous one. The running time of a film is a continuous variable that affects the viewing experience of audience, which may influence word of mouth and subsequent purchase behaviors. Table 1 presents descriptions of the key variables in this study.

## Results

### Multiple Regression Analysis

#### Basic Results

We used STATA 15.0 to perform the analysis, and following the common practice in film studies (e.g., Chintagunta et al., 2010), 0.1, 0.05, and 0.01 significance level is employed. The actual valid sample size is 306. As the issue of endogeneity is not addressed (please see more details in section Endogeneity Corrected Estimations) in this section, the results are only interpreted as associations. A robust ordinary least squares estimation was applied in addition to Equations (1–3) in order to examine the differences between the associations of verified users' and unverified users' online review valences and their variances with online ticket sales (Table 2, Models 1–3). As shown in Table 2, the coefficient of verified users' online review valences is 0.243, whereas that of the unverified users' online review valence is −0.090 (Model 1); only the former reaches the level of significance. The association of the verified users' online review valences with film sales is opposite to that of the unverified users'; that is, consumers are convinced by the verified users' ratings. By contrast, the consumers are skeptical about the unverified users' film ratings; as the unverified users' ratings go higher, the consumers react more negatively to the ratings, which reduces their ticket purchase intentions. It indicates that consumers are able to distinguish identified reviews from unidentified ones, and the unidentified reviews do not convince them to purchase tickets. Specifically, the positive association between verified users' online review ratings and ticket purchases is more significant than that of the unverified users'.

**Table 2:** Results of regressions testing for direct and moderation effects.

**Dependent variable: the total number of online ticket sales**	**Model 1**	**Model 2**	**Model 3**	**Model 4**	**Model 5**
(ln)online customer reviews (volume)	1.081^***^	1.084^***^	1.082^***^	1.087^***^	1.072^***^
	(0.032)	(0.029)	(0.031)	(0.030)	(0.029)
Total valence		0.156^***^			0.161^***^
		(0.058)			(0.057)
Total variance of ratings	0.488^**^			0.465^**^	
	(0.200)			(0.191)	
Valence of verified users' reviews (RURs)	0.243^***^		0.191^***^	0.309^***^	
	(0.078)		(0.071)	(0.087)	
Valence of unverified users' reviews (RNURs)	−0.090		−0.032	−0.236^***^	
	(0.090)		(0.095)	(0.087)	
Variance of verified users' reviews (VURs)		−0.085	−0.026		−0.190
		(0.148)	(0.156)		(0.164)
Variance of unverified users' reviews (VNURs)		0.544^***^	0.517^**^		0.676^***^
		(0.176)	(0.216)		(0.190)
Star power	0.190^***^	0.200^***^	0.188^**^	−0.962^*^	0.574^*^
	(0.072)	(0.075)	(0.073)	(0.511)	(0.338)
RURs*Star power				−0.275^**^	
				(0.129)	
RNURs*Star power				0.431^***^	
				(0.136)	
VURs*Star power					0.828^***^
					(0.220)
VNURs*Star power					−0.926^***^
					(0.239)
**Control variables**					
Follow-up sequel	0.244^**^	0.239^**^	0.239^**^	0.215^**^	0.230^**^
	(0.096)	(0.097)	(0.097)	(0.093)	(0.096)
Released in holiday	0.102	0.109	0.101	0.126	0.137
	(0.117)	(0.119)	(0.118)	(0.114)	(0.118)
**Country of origin**					
Domestic film	−0.083	−0.064	−0.072	−0.111	−0.065
	(0.111)	(0.109)	(0.110)	(0.107)	(0.107)
Foreign film	0.565^***^	0.589^***^	0.584^***^	0.536^***^	0.575^***^
	(0.128)	(0.127)	(0.131)	(0.121)	(0.123)
**Publisher**					
State-owned sector	0.057	0.074	0.065	0.056	0.071
	(0.112)	(0.112)	(0.112)	(0.110)	(0.110)
Large private-sector	0.102	0.126	0.097	0.119	0.137
	(0.125)	(0.125)	(0.126)	(0.124)	(0.122)
**Cinematic genre**					
Story	−0.239^*^	−0.194	−0.225	−0.249^*^	−0.276^**^
	(0.139)	(0.144)	(0.139)	(0.136)	(0.136)
Comedy	−0.128	−0.072	−0.118	−0.137	−0.133
	(0.149)	(0.147)	(0.147)	(0.146)	(0.139)
Actioner	−0.447^***^	−0.431^***^	−0.433^***^	−0.473^***^	−0.496^***^
	(0.143)	(0.148)	(0.145)	(0.143)	(0.140)
Romance	−0.355^*^	−0.266	−0.313^*^	−0.361^**^	−0.357^**^
	(0.182)	(0.186)	(0.182)	(0.180)	(0.176)
Cartoon	0.284	0.350^*^	0.303	0.275	0.257
	(0.198)	(0.190)	(0.196)	(0.193)	(0.182)
Horror	0.375^**^	0.323	0.343^*^	0.305	0.189
	(0.188)	(0.199)	(0.187)	(0.193)	(0.198)
**Screening format**					
2D	0.021	−0.000	0.007	0.013	0.040
	(0.117)	(0.118)	(0.118)	(0.119)	(0.121)
3D	−0.123	−0.128	−0.133	−0.130	−0.096
	(0.117)	(0.119)	(0.119)	(0.122)	(0.121)
IMAX	0.135	0.139	0.133	0.142	0.134
	(0.109)	(0.109)	(0.110)	(0.107)	(0.107)
Length of the film	0.003	0.003	0.002	0.002	0.002
	(0.003)	(0.003)	(0.003)	(0.003)	(0.003)
Number of films in the same week	0.016	0.014	0.018	0.010	0.012
	(0.023)	(0.025)	(0.024)	(0.023)	(0.024)
**Monthly dummies**	Yes	Yes	Yes	Yes	Yes
Constant	0.665	0.665	0.608	1.364^*^	0.690
	(0.931)	(0.754)	(0.900)	(0.777)	(0.785)
R^2^	0.921	0.922	0.922	0.925	0.926
Adj. R^2^	0.911	0.912	0.912	0.916	0.916
OBS	306	306	306	306	306
RMSE	0.620	0.616	0.617	0.603	0.602

*Source: Gewara and China Box-Office, 2013–2014*.

Robust standard errors in parentheses; Sig:

* *p < 0.1*,

** p < 0.05,

*** *p < 0.01*.

The coefficient of the variance of verified users' online reviews is −0.085, whereas that of the variance of unverified users' online reviews is 0.544 (Table 2, Model 2). Only the latter reaches the level of significance. It indicates that the higher variance of unverified users' online reviews is associated with more online ticket purchases on Gewara. The variance among unverified users' online reviews appears to have a significantly stronger association with ticket purchases than the variance among verified users' online reviews.

In terms of control variables, the number of online reviews is significantly correlated with the number of tickets purchased, displaying an awareness effect. Furthermore, the coefficient concerning the status of the films as sequels is significantly positive, signifying that sequels can attract more ticket purchases than non-sequels. That is possibly because sequels are more cost-efficient to develop and market, as it is much easier to make advertising among consumers who have already been familiar with the concept (Eliashberg et al., 2006). Finally, Model 3 shows that simultaneously involving verified users' and unverified users' review ratings and variances only slightly changes the sizes of the coefficients for verified users' review ratings and unverified users' review variances, but the associations between unverified users' review valences or verified users' review variances and online sales remain insignificant.

#### Effect of Brand Strength (Star Power) on Online Ticket Sales

Through Equations (4, 5), this study investigated the moderating effect of brand strength (star power) on the association between verified users' and unverified users' online reviews and the number of tickets purchased online. The interaction term between verified users' review ratings and the star power of the films is significantly negative (Table 2, Model 4). It indicates that the online reviews from verified users may have a significantly weaker association with the number of tickets purchased for the films with strong star power (0.309 – 0.275 = 0.034) than with that of films with weak star power (0.309). By contrast, the interaction term between the unverified users' review valences and the star power of the films is significantly positive. It means that the online reviews from unverified users may have a significantly stronger association with the number of tickets purchased for films with strong star power (−0.236 + 0.431 = 0.195) than with that of films with weak star power (−0.236). Moreover, through the moderating effect of star power, the association between the unverified users' reviews and the number of ticket purchased for the films with strong star power becomes positive; nevertheless, the association of the reviews with the number of ticket purchased for films with weak star power remains negative.

**Figure 2:**
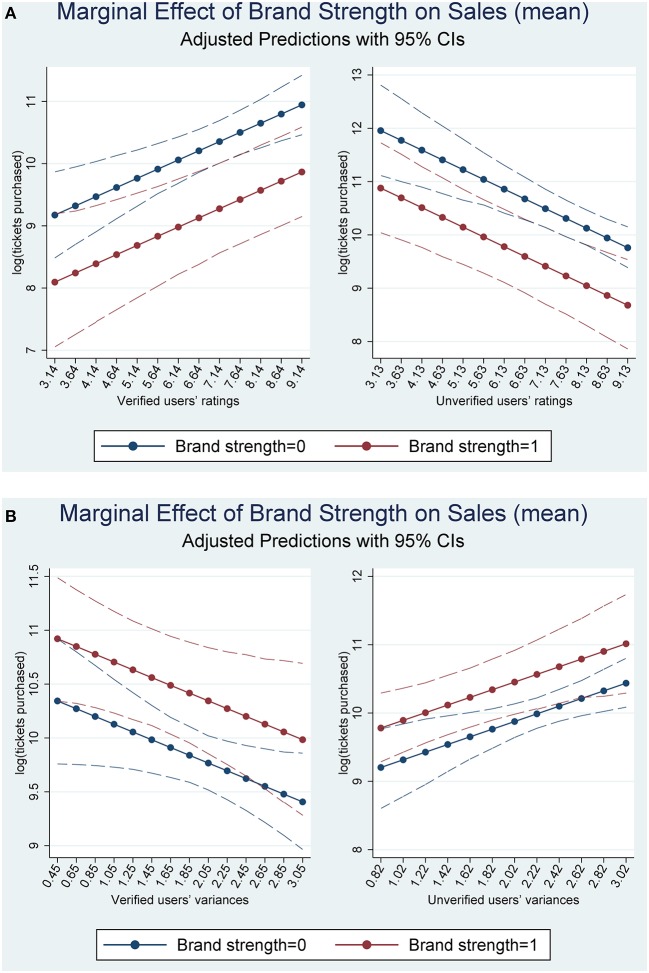
Interactions between online reviews and brand strength. **(A)** Valence and brand strength. **(B)** Variance and brand strength.

The interaction term between the variance of the verified users' reviews and the star power of the films is significantly positive (Table 2, Model 5), which indicates that the variance of the verified users' online reviews may have a significantly stronger association with the number of ticket purchased for the films with strong star power (0.828) than on that for films with weak star power. By contrast, the interaction term between the variance of the unverified users' reviews and the star power of the films is significantly negative. It reveals that the variance of the unverified users' online reviews may have a significantly weaker association with the number of tickets purchased for films with strong star power (0.676 – 0.926 = −0.250) than with that for films with weak star power (0.676). Finally, we provide interaction plots with 95% CIs to show the slopes of these relationships in Figures 2A,B.

### Robustness Analysis

#### Endogeneity Corrected Estimations

Following Holbrook and Addis (2008) and Peng et al. (2019), we develop different simultaneous equation sets to test the sensitivity of our results to the potential endogeneity. Table 3 shows the simultaneous equation estimation of the systems of Equations (4, 5). Column 1 in Table 3 shows the estimation results pertaining to Equation (6) (i.e., Equation 4 in the system), where box-office sales is a dependent variable. The estimated results show that strong brand strength, sequel, valence of verified users' reviews have positive and significant effects on box-office sales; the interactive term (valence of verified users' reviews × strong brand strength) has significantly negative effects, but the impacts of valence of unverified users' reviews and the interactive term (valence of unverified users' reviews × strong brand strength) are statistically insignificant. Columns 2–7 show the estimation results pertaining to Equations (7–12), where the number of online reviews, total variance, valence of verified users' reviews, valence of unverified users' reviews and interactive terms (valence of verified users' reviews × strong brand strength, valence of unverified users' reviews × strong brand strength) are dependent variables. The results show that rating has a negative and significant effect on variance, while strong brand strength affects interactive terms positively and significantly. Column 8 in Table 3 shows the estimation results pertaining to Equation (5) (the first equation in the system). The findings demonstrate that total valence, the variance of verified users' reviews and variance of unverified users' reviews × strong brand strength have negative and significant effects on box-office sales but the impacts of variance of unverified users' reviews and variance of verified users' reviews × strong brand strength are significantly positive. It is found that while strong brand strength continues to reduce the positive effect of verified users' ratings on box-office sales, ratings of unverified users' reviews does not play an important role in the decision-making process. Furthermore, like the earlier results (Equation 5), the impact of variances of unverified reviews is greater than that of verified users, especially when the brand strength is low. We also compared the results based on simultaneous equation setups with the basic results shown in column 1–3, Table 2^7^. The findings show that our basic conclusions do not significantly change even when potential endogeneity is taken into account. Thus, hypotheses H1, H2, H3a, H4a, and H4b are confirmed. The only exceptional case is H3b where the results are sensitive to different model settings.

**Table 3:** Endogeneity control (simultaneous equations).

**Dependent variable: the total number of online ticket sales Model 6**								**Model 7**
	**lnbuy**	**(ln)online customer reviews**	**Variance**	**RURs**	**RNURs**	RURs ^*^Star power	RNURs ^*^Star power	**lnbuy**
(ln)online customer reviews	0.916^***^ (−0.110)							0.970^***^ (0.071)
lnbuy		0.869^***^	−0.072	0.337^***^	0.127^***^	−0.05	−0.001	
		(−0.032)	(−0.021)	(−0.036)	(−0.030)	(−0.026)	(−0.026)	
New week		0.006^**^						
		(−0.002)						
Total valence		−0.064	−0.180^***^					0.319^***^
		(−0.033)	(−0.019)					(0.061)
Holiday		−0.169^**^						
		(−0.061)						
2D		−0.045						
		(−0.090)						
3D		−0.077						
		(−0.106)						
Foreign film		0.164^*^	0.140^*^					
		(−0.072)	(−0.060)					
Large private-sector			0.008					
			(−0.052)					
Small private-sector			0.110					
			(−0.064)					
Actress			0.129^*^					
			(−0.054)					
Variance	−1.536^***^							
	(−0.287)							
Star power	22.08^***^					7.324^***^	7.221^***^	−2.150
	(−3.151)					(−0.079)	(−0.080)	(1.317)
Sequel	0.278^*^							−0.019
	(−0.131)							(0.102)
Length of the film	0.008							0.002
	(−0.005)							(0.004)
Story	−0.083							0.001
	(−0.094)							(0.063)
Romance	−0.008							−0.001
	(−0.083)							(0.073)
Comedy	0.151							0.012
	(−0.113)							(0.082)
Actioner	−0.093							0.041
	(−0.106)							(0.075)
Horror	−1.151							−0.057
	(0.003)							(0.084)
Cartoon	−0.005							0.071
	(−0.149)							(0.107)
Valence of verified users' reviews (RURs)	0.660^***^ (−0.178)					0.300^***^ (−0.028)		
Valence of unverified users' reviews (RNURs)	−0.255 (−0.177)						0.264^***^ (−0.033)	
Variance of verified users' reviews (VURs)				−1.258^***^ (−0.098)				−2.017^***^ (0.288)
Variance of unverified users' reviews (VNURs)					−1.493^***^ (−0.082)			1.537^***^ (0.316)
RURs*Star power	−2.997^**^							
	(−1.151)							
RNURs*Star power	0.003							
	(−1.079)							
VURs*Star power								8.428^***^ (1.275)
VNURs*Star power								−6.352^***^ (1.418)
cons	3.244^**^	−2.193^***^	3.778^***^	6.188^***^	9.057^***^	−1.639^***^	−1.903^***^	1.789^*^
	(−1.091)	(−0.361)	(−0.201)	(−0.451)	(−0.403)	(−0.223)	(−0.238)	(0.878)
R^2^	0.25	0.87	0.46	0.46	0.97	0.97	0.78	0.62

*Source: Gewara and China Box-Office, 2013–2014*.

Robust standard errors in parentheses; Sig:

* *p < 0.1*,

** *p < 0.05*,

*** *p < 0.01*.

#### Robustness Test for Different Proxies of the Box-Office Dependent Variable

Because Gewara is a ticket purchase website that provides information on online reviews and ticket purchases, the aforementioned analyses (Table 2, Models 5 and 6) are conducted again by using the number of tickets purchased through Gewara as the dependent variable. To further investigate the external validity of the effects of the verified users' and unverified users' online reviews on the number of purchased tickets, we have analyzed the effects of these online reviews on consumer attitudes (likes and attention), the performance of the website (conversion rates), and the performance of the companies (the total box-office sales and the box-office sales that excludes the first-week sales), as presented in Tables 4, 5.

**Table 4:** Robustness test for the different dependent variable (Model 8).

**Dependent variable**	**Tickets**	**Box-office (1)**	**Box-office (2)**	**Like**	**Attention**	**Conversion rate**
(ln)online customer reviews	1.087^***^	0.873^***^	0.939^***^	0.992^***^	0.694^***^	0.079^***^
	(0.030)	(0.042)	(0.047)	(0.027)	(0.021)	(0.004)
Valence of verified users (RURs)	0.309^***^	0.251^**^	0.239^*^	0.237^***^	0.177^***^	0.021^**^
	(0.087)	(0.102)	(0.125)	(0.077)	(0.061)	(0.009)
Valence of unverified users (RNURs)	−0.236^***^	−0.357^***^	−0.321^**^	−0.142^*^	−0.139^**^	−0.012
	(0.087)	(0.104)	(0.124)	(0.078)	(0.060)	(0.010)
Total variance of ratings	0.465^**^	0.536^**^	0.484^**^	0.472^***^	0.299^**^	0.035^*^
	(0.191)	(0.218)	(0.242)	(0.172)	(0.118)	(0.019)
RURs*Star power	−0.275^**^	−0.434^**^	−0.297	−0.185	−0.125	−0.018
	(0.129)	(0.201)	(0.230)	(0.123)	(0.093)	(0.016)
RNURs*Star power	0.431^***^	0.479^**^	0.413^*^	0.319^**^	0.261^**^	0.032^**^
	(0.136)	(0.198)	(0.240)	(0.129)	(0.105)	(0.015)
Constant	1.364^*^	2.217^**^	1.066	0.711	5.634^***^	−0.311^***^
	(0.777)	(0.936)	(1.027)	(0.709)	(0.519)	(0.087)
All controls	Yes	Yes	Yes	Yes	Yes	Yes
R^2^	0.925	0.828	0.807	0.929	0.933	0.761
Adj. R^2^	0.916	0.806	0.782	0.920	0.925	0.730
OBS	306	306	306	306	306	306
RMSE	0.603	0.774	0.911	0.539	0.376	0.084

*Source: Gewara and China Box-Office, 2013–2014*.

Robust standard errors in parentheses; Sig:

* *p < 0.1*,

** *p < 0.05*,

*** *p < 0.01*.

*Box-office (1) is the total box-office sales, Box-office (2) is the box-office sales that excludes the first-week sales*.

**Table 5:** Robustness test for the different dependent variable (Model 9).

**Dependent variable**	**Tickets**	**Box-office (1)**	**Box-office (2)**	**Like**	**Attention**	**Conversion rate**
(ln)online customer reviews	1.072^***^	0.842^***^	0.923^***^	0.980^***^	0.685^***^	0.077^***^
	(0.029)	(0.039)	(0.046)	(0.026)	(0.019)	(0.004)
Variance of verified users (VURs)	−0.190	−0.080	−0.144	−0.122	−0.125	−0.007
	(0.164)	(0.209)	(0.233)	(0.141)	(0.100)	(0.016)
Variance of unverified users (VNURs)	0.676^***^	0.928^***^	0.862^***^	0.557^***^	0.483^***^	0.046^**^
	(0.190)	(0.215)	(0.250)	(0.169)	(0.109)	(0.019)
Total valence	0.161^***^	0.136^**^	0.125^*^	0.144^***^	0.139^***^	0.014^***^
	(0.057)	(0.067)	(0.071)	(0.049)	(0.034)	(0.005)
VURs*Star power	0.828^***^	1.045^*^	0.513	0.656^***^	0.408^***^	0.086^***^
	(0.220)	(0.554)	(0.498)	(0.203)	(0.142)	(0.028)
VNURs*Star power	−0.926^***^	−0.909^*^	−0.495^*^	−0.753^***^	−0.526^***^	−0.113^***^
	(0.239)	(0.495)	(0.506)	(0.214)	(0.142)	(0.029)
Constant	0.690	0.188	−0.726	0.421	4.840^***^	−0.352^***^
	(0.785)	(0.947)	(0.997)	(0.686)	(0.500)	(0.079)
All controls	Yes	Yes	Yes	Yes	Yes	Yes
R^2^	0.926	0.830	0.807	0.930	0.938	0.766
Adj. R^2^	0.916	0.808	0.782	0.921	0.930	0.736
OBS	306	306	306	306	306	306
RMSE	0.602	0.770	0.909	0.536	0.363	0.083

*Source: Gewara and China Box-Office, 2013–2014*.

Robust standard errors in parentheses; Sig:

* *p < 0.1*,

** *p < 0.05*,

*** *p < 0.01*.

*Box-office (1) is the total box-office sales, Box-office (2) is the box-office sales that excludes the first-week sales*.

Overall, the results are highly robust across model specifications. The verified users' online review valences significantly and positively influence box-office sales as well as consumers' likes and attention, thus improving purchase conversion rates. With the exception of conversion rate, the effects of the unverified users' online review valences on the dependent variables are all negatively significant. The variance of the verified users' online reviews exerts negative and insignificant effects on all the dependent variables; and the variance of the online reviews from the unverified users significantly and positively influences all dependent variables. Most importantly, the interactive effects between these online reviews and brand strength are similar to the previous results.

## Discussion and Conclusions

### Discussion

The effect of online reviews on consumers' purchase intentions and behaviors has long been known by scholars. However, the present study employs “verified users” and “unverified users” as labels for factual verification to explore the influence mechanism of information exchange and acquisition while conceptualizing a theoretical framework based on the psychological choices model concerning how source identity enhances source credibility. Within this theoretical framework, several psychology theories are introduced to develop research hypotheses, such as the heuristic–systematic model (HSM) and the information adoption model (IAM). The findings of this study support the view that features of online reviews and consumers' perceptions of online reviews have a substantial impact on consumers' purchase intentions which will further affect their purchase behaviors. Specifically, our findings support **Hypothesis 1** that verified users' online review ratings exert a significantly more positive effect on the number of purchases than non-verified users' online review ratings do. This is consistent with the findings of previous studies, which suggest that reviews with identified sources have a greater effect on forming consumer perceptions of review credibility than unidentified ones do (Wathen and Burkell, 2002; Stiff and Mongeau, 2016). Building on this, and with regards to **Hypothesis 2**, in this study we further explored the differential effect of variance of ratings. When the impact of unverified users' reviews is examined in terms of variance, such reviews are likely to positively influence the number of purchases. This finding is an important contribution to the literature, because most studies (e.g., Xie et al., 2011; Ladhari and Michaud, 2015) have only suggested that identified sources are determinants of persuasion and market outcome. An unreliable high variance of ratings may imply the hype effect of a given movie (Reddy et al., 2012) which is produced by a “positive/negative opinion spam” by rival promotion companies or consumers' watch dog behaviors toward such an abnormal, polarized ratings (Trope and Liberman, 2010). In either way, the distribution of these unverified ratings would be greatly widened which in turn significantly increases the awareness of a movie and also arouses much more potential consumers' interests.

On the other side, after considering consumer behaviors toward online reviews in general, we have included brand strength into the analysis. The results generally confirmed **Hypothesis 3** and **Hypothesis 4**, as the brand strength of a movie product moderates the effects of online reviews on box-office performance in quite dynamic ways under different conditions. With this in mind, and in accordance with **Hypothesis 3(a)**, the findings further suggest that the positive effect of verified users' online review valences on the number of tickets purchased for films decreases in association with high brand strength. Therefore, in terms of box-office sales, the effect of verified users' online review valences should not be viewed as its own as it is related to the power of brand strength too. With a higher level of brand strength, consumers would feel a brand will take care of uncertain issues in an unforeseeable circumstance, such as buying a ticket for a new movie (Chang et al., 2013). The only exception is **Hypothesis 3(b)**, as the result did not pass the robustness test. This indicates that no significant relationship has been found between unverified users' online review ratings and the number of tickets purchased through interpreting the strength of a brand. One possible cause of this result is that the exploratory power of verified users' online review valences, in association with the effect of brand strength, is much stronger than those of unverified users'. As a result, its effect was suppressed in the endogeneity corrected model. However, this finding does not amend the conclusion that ratings with identified sources have a greater effect on forming consumer perceptions of review credibility than unidentified ones do.

Moreover, with regards to **Hypothesis 4(a)**, we find that the variance of verified users' online reviews positively affects the number of tickets purchased for films with high brand strength, but such an effect is negative with low brand strength. This finding means that consumers have higher brand trust toward a strong brand, thus in an association with the effect of identified sources, they are more likely to believe the distribution of ratings is truthful, and an easy observation of a truthful high variance of ratings would facilitate in matching consumers' expectations and movie productions (Clemons et al.,2006; Sun, 2012). However, on the other hand, the confirmation of **Hypothesis 4(b)** suggests that the variance of unverified users' online reviews positively influences the number of tickets purchased for films with low brand strength, but it negatively influences the number of tickets purchased for films with high brand strength. This finding implies information authenticity is not an absolute benchmark to distinguish the positive or negative effect of online reviews for a movie. At least in the case of “low brand strength and unverified variance,” Chinese consumers appear to believe that buying a ticket could satisfy their epistemic curiosities (Litman, 2008) that eliminate information gaps while less considering the reliability dimension of brand trust (Deighton, 1992). Therefore, it has been proved that not all online information has the same effect on consumer's purchase intentions and behaviors (Yang, 2012); the level of impact varies according to different information characteristics. Sometimes, consumers would go beyond information they have been given, and subtle environmental cues can activate associated subjective representation of the importance or perceived risk of a product (Laurent and Kapferer, 1985).

### Organizational Implications

The present study simulates a realistic marketing environment by suggesting that source identity and brand strength play crucial roles in forming consumers' perceptions of online review credibility and subsequent purchase behaviors. Even though these findings do not directly deal with human behavior in organizations, considering that organizations are human creations that consists of people who influence each other (Hewstone and Stroebe, 2001), the perspective of this study still improves the understanding of various themes dealing with organizational viability or performance, such as strategic mission.

Our research framework shows that the underlying mechanisms of consumers' online behaviors in China are much more complicated than we thought in the context of movie product. Chinese consumers might be less likely to provide extremely negative reviews, as many of previous studies suggested (e.g., Fang et al., 2013). However, they are still very sensitive to some perspectives of online reviews, including review's quality and review's feature, and subsequent reactions and market outcomes vary from case to case. Thus, it is important for Chinese movie companies to allocate more resources to managing different types of online customer reviews. Moreover, the study has also shown that the presence of source identity and brand strength in an online communication environment can affect the persuasiveness of online reviews. However, it appears that there is no a “one-size-fits-all” strategy to stimulate consumer's purchase intentions and behaviors. It is better for business leaders to understand not only why producers of online reviews are satisfied or dissatisfied, but also how consumers interpret and interact with different types of online reviews and which are important. This requires a smart and flexible collaboration among different business units within film company. For instance, promotion managers should respond rapidly to the numerous ratings from unverified users that amplify the celebrity effect and brand effect of products, in association with the effort of IT team on designing systems to make verified ratings available for reviewers. It is also possible that IT team should only limit the disclosure of high variance of ratings from unverified users if the brand strength is considered as high. When marketing department defines the brand strength of its film is lower than competitors', IT team however, should expose a high variance of unverified ratings to curious consumers. In other words, film business has to acknowledge that dealing with online reviews is not only a technical issue; it can have a substantial effect on organizational performance through several dimensions (e.g., fake vs. real or brand vs. hype) in the dynamic online communication environment.

Although these findings have made meaningful contributions to the studies in the field of online reviews, the present study still has several limitations. First, this study takes films as the research object. Although products like books, performance, and audio CDs are similar to films, no relevant data were collected for verifying the external validity (generalizability) of the study results. Therefore, future studies must be carried out according to the conclusions of this study. Second, the difference between the effects of verified users' and unverified users' online reviews on product sales should be investigated from the perspective of their textual features. Third, the differences in authenticity among online reviews posted via different channels should be compared, and the effects of such reviews on consumer attitudes, intentions, and behaviors should be analyzed. Last, the effect of malicious reviews on the welfare of consumers, companies, and society needs to be verified.

## Data Availability Statement

The raw data supporting the conclusions of this article will be made available by the authors, without undue reservation, to any qualified researcher.

## Ethics Statement

This study was approved by the Research Ethics Committee at the North China University of Technology. All data were obtained from publicly accessible resources at the aggregate level. The study and protocol were also reviewed and approved by the School Sub-committee under the Research Ethics Committee at the North China University of Technology.

## Author Contributions

KZ and XY developed the concept and design of this study. XY, XT, and JZ performed the experiments and analyzed the data. XY, XX, and KZ wrote the paper. KZ restructured, polished, and revised the paper.

### Conflict of Interest

The authors declare that the research was conducted in the absence of any commercial or financial relationships that could be construed as a potential conflict of interest.

## Footnotes

^1^These publicly accessible datasets are commonly used in Chinese movie studies. For instance, Peng et al. (2019) used these data to construct a secondary motive database over the period 2009–2016.

^2^It is possible to develop a measure by counting the number of awards that different people involved have won for each film. However, the effect of cast size would lead to an inaccurate estimation of star power in general. For example, maybe there is only one very famous actor who has won five awards in movie A, while there are six rookie mover stars in movie B and each of them just won one award. Measuring the star power by counting the total awards gives a conclusion that the star power of movie A is weaker than B, but consumers would be less aware of such a difference and the existing dataset is difficult to tell whether consumers care more about just 1 or 2 movie stars in the cast or all of them. Moreover, it should be pointed out that the casts and directors of many Chinese movies haven't won any film award. If the brand strength is measured by the number of awards, it leads to the issue of missing data. Therefore, it is practical to measure the star power of a movie from a perspective of “general familiarity,” as many branding researches suggest.

^3^http://www.gewara.com/

^4^http://www.cbooo.cn/

^5^It is ideal to collect more data from Gewara, but due to the rapid development of online ticketing services in China, a number of influential online ticketing platforms have emerged since 2015. Therefore, the present study only focuses on the period from January 2013 to October 2014 and there are two reasons: first, Gewara was the most influential online ticketing platform during this period of time (Lee, 2012; Zhu et al., 2016), so consumers are less likely to purchase movie tickets online from other platforms; second, the corresponding box-office sales is therefore more likely to reflect market outcomes of consumers' behaviours on Gewara. Moreover, we only selected movies on the top 20 of the national box-office sales in each show period, thus the data of 308 movies were collected in total. Gewara does not provide sufficient data of movies with box-office sales rank outside top 20, as they are usually made on shoestring budgets, and have very limited number of screenings.

^6^The multicollinearity test for the basic models 1–3 indicates that all VIF values of the variables are below 10, and the signs and magnitudes of variable coefficients based on the basic models do not significantly change with the further involvement of interactive terms, therefore we believe that our model specification is less likely to have multicollinearity problem.

^7^Limited by the length of the paper, the econometric results of Equations (1)–(3) are not presented here.
